# Effect of Controlled Oxygen Supply during Crushing on Volatile and Phenol Compounds and Sensory Characteristics in Coratina and Ogliarola Virgin Olive Oils

**DOI:** 10.3390/foods12030612

**Published:** 2023-02-01

**Authors:** Gianluca Veneziani, Diego L. García-González, Sonia Esposto, Davide Nucciarelli, Agnese Taticchi, Abdelaziz Boudebouz, Maurizio Servili

**Affiliations:** 1 Department of Agricultural, Food and Environmental Sciences, University of Perugia, Via S. Costanzo, 06126 Perugia, Italy; 2 Instituto de la Grasa (CSIC), Ctra. Utrera, km 1, Edif. 46, 41013 Sevilla, Spain; 3 Instrument Sensometry Research Group (i-Sens), Department of Analytical Chemistry and Organic Chemistry, Universitat Rovira i Virgili, Campus Sescelades, 43007 Tarragona, Spain

**Keywords:** virgin olive oil, crushing, volatile compounds, phenols, oxygen control

## Abstract

In virgin olive oil industries, the technological choices of the production plant affect the biochemical activities that take place in the olives being processed throughout the entire process, thereby affecting the quality of the final product. The lipoxygenase pool enzymes that operated their activity during the first phases of the process need the best conditions to work, especially concerning temperature and oxygen availability. In this study, a system was equipped to supply oxygen in the crusher at a controllable concentration in an industrial olive oil mill at pilot plant scale, and four oxygen concentrations and two cultivars, Coratina and Ogliarola, were tested. The best concentration for oxygen supply was 0.2 L/min at the working capacity of 0.64 Ton/h. Further, using this addition of oxygen, it was possible to increase the compound’s concentration, which is responsible for the green, fruity aroma. The effect on volatile compounds was also confirmed by the sensory analyses. However, at the same time, it was possible to maintain the concentration of phenols in a good quality olive oil while also preserving all the antioxidant properties of the product due to the presence of phenols. This study corroborates the importance of controlling oxygen supply in the first step of the process for process management and quality improvement in virgin olive oil production.

## 1. Introduction

The sensory characteristics of extra virgin olive oil (EVOO) are very relevant to the choice that the customer makes at the moment of purchasing the product. Thus, consumers have been gaining knowledge of the different desirable sensory notes found in virgin olive oil in the last few years, and producers are consequently adapting production to a higher quality [1,2,3,4,5,6,7]. In order to improve quality, producers are collecting in the right ripening period to prioritize quality in addition to yield [8,9,10,11,12]. Furthermore, the contribution of the different cultivars is carefully handled in addition to other agronomical factors [7,13,14] and commercialization variables [1,3]. Thus, all these factors affect the volatile composition [4,13,15,16] and, therefore, the aroma of the product. In addition to the sensory attributes, consumers are also aware of the health benefits associated with virgin olive oil consumption, and they attribute these benefits to the presence of phenols, among other compounds. The phenols are also relevant in EVOO because of their sensory impact, contributing to bitterness and pungency. Thus, it is important to develop and improve the sensory properties of EVOO while maintaining its health characteristics, in order to have a pleasant product that is at the same time also healthy. That means that all the processes during production and distribution involving lipid oxidation may affect volatile and phenol concentrations, and therefore they need to be carefully addressed and optimized.

In the optimization of virgin olive oil quality, the different variables associated with the mechanical methods used for oil extraction have to be considered since they affect virgin olive oil quality and the content of phenols and volatiles [17]. In particular, a lot of interest has been put into the first phases of EVOO production. In addition, several studies have been addressed with the aim of improving quality, especially in the last few years, using new technologies or optimizing the variables that affect EVOO composition [18,19,20,21,22]. Further, the interest of the research activities was focused on the initial part of the process, starting from the malaxation phase, in which temperature, time, and oxygen play a significant role in the development of chemical and biochemical reactions influencing the quality of the final product [18,22,23,24]. Notably, the thermal conditioning of the olive paste by means of a heat exchanger prior to malaxation has proven to be of paramount importance for managing the enzyme activities responsible for the production of volatile compounds and the oxidation of phenols [18,22,25]. Recently, other technologies were imported into the olive oil sector, coming from other food industries. This is the case for ultrasound, pulsed electric field, and high vacuum malaxation. These technologies, when applied to the production of virgin olive oil, are able to increase the quality and the extraction yield in different ways. All these technologies have the purpose of freeing the oil from the vacuoles and improving the separation of phases during malaxation in order to have the best condition before extraction [20,21,26,27,28]. However, not only the yield extraction is the main interest of applying these technologies, but also the quality improvement, for example, due to the rise in phenol concentration and the production of oils with healthier properties and an extended shelf life [19,28,29,30]. The increase in phenol concentration is due to the fine cellular breaking and the freeing of the cytoplasmatic liquid, thus promoting the contact between oil droplets and all the other minor compounds [31,32,33].

In addition to the work focused on improving the malaxation step, research has recently been focused on the crushing step. In addition, the preparation of the paste for the malaxation is initially made with the crushing of the olives, which is the first mechanical process of the fruits, and it has a fundamental role in starting all the biochemical processes [34,35,36]. However, the degree of innovation in this step is relatively lower compared with other stages in the production, mainly malaxation. A relevant early innovation was the use of a hammer crusher as an alternative to stone crushers, which was later coupled with a continuous centrifuge system [17]. The latter allowed a higher degree of automatization to permit a simple management of the process and enhanced quality [37]. Furthermore, the innovation in olive crushing continued with the invention of new cutting elements for the crushers, such as knife crushers and disk crushers. The purpose of this innovation was to perform a differentiated crushing that considers the location of the endogenous enzymes in the olive fruit [38]. This differentiated crushing resulted in an improvement in the quality, related to an increase in concentrations of volatile compounds and phenols. One of the latest innovations in crushing is the “double effect crusher”, which is a mixed system between a disk crusher and a hammer crusher [39]. It maintains the principle of differentiated crushing explained above, but it can also produce a more homogeneous olive paste by using a sieve located externally to the disks [39]. The thermal conditioning during the crushing phase was also studied as an innovation to avoid the rise in temperature that normally happened due to the high quantity of kinetic energy given by the hammers to the olives that put up the temperature of the olive paste at 5 °C to 10 °C higher than the temperature of the olives before crushing [40].

Previous works implementing modification in production have demonstrated the importance of oxygen in the enzyme activities and in their effect on quality. However, the quantity of oxygen during the malaxation phase has been modulated [41]. In addition, when higher oxygen quantities were supplied, the concentration of volatile compounds increased, and, at the same time, it was observed that oxygen may oxidize phenolic compounds at different degrees according to malaxation temperature and cultivar differences. Other studies showed that the maximum activity of the lipoxygenase pool enzymes takes place in the initial part of the process while olive fruits are breaking up [4,42,43,44]. Thus, these studies evidence that it is important to evaluate the effect of different parameters regulating the activities of the lipoxygenase pathway [45,46]. There is a strong interest in understanding how the parameters of the technological process should be managed to obtain the desired results, as is typically the case in the food industry, in which all the production processes are strictly managed, especially in those cases in which biotechnological activities are involved [47]. Thus, from the point of view of EVOO production, the operating conditions should permit the best quality possible according to the specific enzymes’ activities, which are dependent on the cultivars of the olive fruits [7,22,23,48]. With this purpose, much research has addressed the study of the main factors that influence the activity of lipoxygenase enzymes, such as optimal working temperature, oxygen availability, type and concentration of fatty acids in the olive, and gene expression that modulates protein production [49,50,51,52,53].

Furthermore, given the importance of oxygen and the optimization possibilities of the crushing step, this work focused attention on testing a new configuration to control oxygen during olive crushing and to examine the effect of the different variables on the volatile and phenolic composition. With the purpose of evaluating the impact on oil quality when this configuration is implemented in a real production system, the study was carried out in an industrial pilot plant. In particular, the aim of this work consists on the controlled addition of an oxygen flow at different concentrations inside the crusher chamber in which the fruit breakage takes place. Two specific cultivars of commercial importance were used in this study to evaluate the cultivar effect. Thus, this work was centered on Coratina and Ogliarola virgin olive oils, and the results were compared between these two cultivars.

## 2. Materials and Methods

### 2.1. Extraction Pilot Plant

The trials were carried out by processing Ogliarola and Coratina cultivars harvested at the end of October and in the first week of November (2019) at medium-early and early ripening stages, respectively. Olive fruits (1800 kg of each cultivar) were harvested from the same plot of land (Puglia region, Italy) and processed within 48 h. The ripening indexes, 0.93 for Coratina and 1.57 for Ogliarola olive fruits, were determined according to the method described by Beltrán et al. [54].

The extraction plant for the production of EVOOs was composed of the following parts:

- Defoliator and washing machine (Mori TEM, Tavarnelle Val di Pesa, Italy);
- Hammer crusher mod. TEM 200 (Mori TEM, Tavarnelle Val di Pesa, Italy) equipped with a flow meter with a sensitivity of ±0.05 L/min only usable for oxygen gas (TEKNOM a/m 145, Figline e Incisa Valdarno, Italy) for oxygen measurement and addition in crushing;
- TCM ViscoLineTM XS, a viscous paste heat exchanger for rapid thermoconditioning of olive paste (Alfa Laval Corporate AB, Monza, Italy);
- Malaxer module, 200 kg (Mori TEM, Tavarnelle Val di Pesa, Italy);
- Decanter oliomio (Mori TEM, Tavarnelle Val di Pesa, Italy) at 300 kg/h;
- Vertical centrifuge separator Alfa Laval UVPX 305 AGT 14 (Alfa Laval S.p.A., Tavarnelle Val di Pesa, Italy).

The concentration of oxygen must be related to the extraction plant capacity, in particular the crusher capacity. In all the tests, the flow of olives at the inlet of the crusher was near 640 kg/h. As a result, the quantity of oxygen was 0.019 L for each kg of olives at an addition flow of 0.2 (L/min), 0.038 L for each kg of olives at an addition flow of 0.4 (L/min), and 0.057 and 0.076 L for each kg of olives, respectively, at 0.6 and 0.8 (L/min) of oxygen in addition during crushing. Thus, the quantities of oxygen that were tested during crushing were 0.019 L, 0.038 L, 0.057 L and 0.076 L supplied at addition flows of 0.2 L/min, 0.4 L/min, 0.6 L/min, and 0.8 L/min, respectively. The oxygen was put inside the crusher chambers using a 1/4” ball valve at a distance of 170 mm from the axle of the crusher, inside the sieve.

After the crushing phase, the olive paste was pumped to the viscous paste heat exchanger at a temperature of 18 °C. Then, the olive paste merged into the malaxer at 25 °C for 30 min of malaxation. The malaxer was filled to its maximum level and hermetically sealed from the outside atmosphere. The filling time was approximately 19 min. After malaxation, the oil was separated using a decanter centrifuge. The feed flow to the decanter was 200 kg/h of olive paste. The oil was finally purified from water, solids, and colloids by the vertical separator centrifuge. The samples were withdrawn at the halfway point of the malaxer’s discharge to avoid contamination with the previous sample for each trial.

### 2.2. Analysis of Legal Quality Parameters

The analyses concerning legal quality parameters relative to free acidity [55], peroxide value [56], and spectrophotometric constants [57] were carried out, and the interpretation was carried out according to regulation and following editing [58].

### 2.3. Analysis of Phenolic Compounds

The extraction and HPLC analyses of phenols were carried out according to Antonini et al. [59], using a Spherisorb ODS1 250 mm × 4.6 mm column with a particle diameter of 5 m (Waters, Milford, MA, USA). The HPLC equipment was composed of an Agilent Technologies 1100 series LC system (Agilent Technologies, Palo Alto, CA, USA). The unit was composed of the following parts: a quaternary pump with vacuum degasser; a thermostatic column section; a UV-Vis (DAD) photodiode detector; and a fluorescence detector (FLD) (Agilent Technologies). The management of all the parts of the equipment and the processing of the chromatographic data were carried out with ChemStation Rev. A. 10.02 (Agilent Technologies, Palo Alto, CA, USA). The oleuropein and ligstroside derivatives [the dialdehydic forms of decarboxymethyl elenolic acid linked to hydroxytyrosol (3,4-DHPEA-EDA or oleacein) and to tyrosol (*p*-HPEA-EDA or oleocanthal), 3,4- (dihydroxyphenyl)ethanol elenolic acid (3,4-DHPEA-EA or an isomer of the oleuropein aglycon), and *p*-(hydroxyphenyl) ethanol elenolic acid (*p*-HPEA-EA or ligstroside aglycon)] and lignans [(+)-pinoresinol and (+)-1-acetoxypinoresinol] were separated and purified from VOO following the method described by Selvaggini et al. [41]. The separation of phenolic compounds was carried out by semipreparative HPLC, conducted using an HPLC system composed of a ternary pump, a diode array detector (DAD), and a workstation (Varian, Walnut Creek, CA, USA), employing a Whatman Partisil 10 ODS-2 column (500 mm × 9.4 mm i.d.). The chemical structures of secoiridoids, derivatives, and lignans were verified by nuclear magnetic resonance spectroscopy (NMR), using the same operating conditions reported in a previous paper, by recording **^1^**H and **^13^**C spectra [60]. The purified compounds were used as standards for HPLC analysis. Tyrosol and hydroxytyrosol were purchased from Cabru s.a.s. (Arcore, Milan, Italy) and Fluka (Milan, Italy). The quantity evaluation of phenolic compounds was made using calibration curves for each compound. The results were expressed as a concentration of mg/kg of virgin olive oil.

### 2.4. Analysis of Volatile Compounds

The quantitative and qualitative analysis of volatile compounds in EVOOs was performed by headspace-solid phase microextraction (HS-SPME), followed by gas chromatography-mass spectrometry (HS-SPME-GC/MS). The sampling of headspace volatile compounds by SPME was applied as follows: Three grams of VOO were placed in a 20 mL vial and tightly capped with a polytetrafluoroethylene (PTFE) septum. The internal standard (2-methyl propylacetate) was added to the sample at a concentration of 9.8 mg/kg. The vial was left for 10 min at 35 °C to allow for equilibration of the volatiles in the headspace. After equilibration, the SPME fiber (a 50/30 μm divinylbenzene/carboxen/polydimethylsiloxane (DVB/CAR/PDMS) with a length of 2 cm (StableFlex, Supelco, Inc., Bellefonte, PA, USA) was exposed to the vapor phase for 30 min to sample the volatile compounds. Then, gas chromatography-mass spectrometry analysis was performed.

The analyses of the volatile compounds were carried out with an Agilent Technologies GC 7890B equipped with a “Multimode Injector” (MMI) 7693A (Agilent Technologies, Santa Clara, CA, USA) and a thermostated PAL3RSI120 autosampler equipped with a fiber conditioning module and an agitator (CTC Analytics AG, Zwingen, Switzerland). The detection system was an Agilent 5977B single quadrupole GC/MSD with an EI Extractor (XTR) source (Agilent Technologies, Santa Clara, CA, USA). The volatiles adsorbed by the fiber were thermally desorbed in the hot GC injector port, which was set in splitless mode, for 5 min at 250 °C. The volatile compounds were separated on a DB-WAXetr column (50 m, 0.32 mm i.d., 1 μm film thickness) (Agilent Technologies, Santa Clara, CA, USA) using helium as the carrier gas at a constant flow rate of 1.7 mL/min. The GC oven heating program started at 35 °C; this temperature was held for 4 min, then increased to 150 °C at a rate of 4 °C/min, increased to 180° C at a rate of 8 °C/min, held for 2 min, increased to 210 °C at a rate of 11 °C/min, and this temperature was held for 13.77 min. The total analysis time was 55 min. The temperature of the transfer line was fixed at 215 °C. The MSD was operated in electron ionization (EI) mode, with an ionization energy of 70 eV, in scan mode, with scanning in the mass range of *m*/*z* 25–350 a.m.u. at a scan rate of 4.3 scan/s and in SIM mode for improving the detection limits. The MS source and the MS quad temperatures were 190 °C and 150 °C, respectively. The volatile compounds were identified by comparing their mass spectra and retention times with those of authentic reference compounds and with the spectra in the NIST 2014 mass spectral library. The volatile compounds were quantified using calibration curves for each compound by internal standard calculation, and the results were expressed in μg/kg of oil.

### 2.5. Sensory Analysis

Sensory analyses were made by nine expert panelists from the Unit Research of Food Technologies of the Department of Agricultural, Food, and Environmental Sciences at the University of Perugia. All the panelists were specifically trained for virgin olive oil sensory analysis [61].

The room for the panel test had a separate position for each panelist equipped with the following elements: samples, a descriptive-quantitative form for sensory analysis (the QDA^®^ method), and oral cleaning devices to be used between the different tests (including sparkling water and apples). The samples were put in white plastic cups and identified by a code of three numbers. Random sequences of samples were given to each panelist in a balanced order to avoid the influence of the results due to the tasting order.

The evaluations were made in a single test by using a descriptive-quantitative form for the sensory analysis of a structured geometric type according to UNI ISO WD 4121 2001. The descriptors were divided into the following categories: color, olfactory characteristics, gustatory-retronasal characteristics, and final olfactory-gustatory sensations, typical of virgin olive oil.

Twenty-one attributes were evaluated: four were for the visible evaluation for the typology of green, eight referred to the smell sensations, and four were related to the gustative perception. In the form there were also some identifiers for the off flavors.

The sensory profile for each sample was reported using a spider graph, and the values were processed using the PCA method to discriminate the samples according to the different parameters considered in the panel test.

### 2.6. Statistical Analysis

The analysis of variance, a one-way ANOVA, was applied with the purpose of identifying the significance of the differences between the data due to the oxygen addition. The means were compared using the Tukey test with a *p* < 0.05. SigmaPlot V. 12.3 was used to process the data (Systat Software Inc., San Jose, CA, USA).

PCA for multivariate statistical elaboration of data was conducted using Panel Check 1.4.2. version software.

## 3. Results and Discussion

### 3.1. Legal Quality Parameters

The extracted virgin olive oils were analyzed according to the legal quality parameters in order to have some basic information about quality. The objective was to check if the sample produced by using different additions of oxygen during the crushing phase could be classified as extra virgin olive oil according to the EU regulation [58]. Table 1 shows the values for free acidity, peroxide value, K232, and K270 for the Ogliarola and Coratina virgin olive oils.

The results showed that the supply of oxygen allowed the production of virgin olive oil within the extra virgin category. In fact, when comparing the values from the virgin olive oil considered the control (no supply of oxygen) and the oils with different values of oxygen flows (0.2–0.8 L/min), it was observed that there were no significant differences associated with the oxygen treatment in all the parameters for the Coratina samples (Table 1). In Ogliarola samples, no significant differences were observed in free acidity, peroxide value, or K270. However, a slight change in K232 was observed in these oils. Thus, K232 fluctuated between 1.709 and 1.841, although with no clear correlation with oxygen flow. In the case of ΔK, the mean value was −0.002 in all the oils except for the sample extracted with 0.8 (L/min) of O_2_, in which the value was −0.004. Oils from cv. Ogliarola showed higher values for all the examined analytical indices compared to the oils obtained from cv. Coratina. These results showed that the oxygen flow addition did not produce a noticeable effect on the legal quality parameters studied, and the flow of oxygen addition was not related to the slight changes identified. The effect on the legal quality parameter should be better investigated in order to identify possible relationships depending also on cultivar and maturity index.

Although the presence of a higher percentage of oxygen could induce lipid oxidation in the olive paste, micro-oxygenation during pressing did not determine a clear effect even on the peroxide value, a parameter that is closely related to oxidation. For both cultivars, in fact, the peroxide values of the control oils were similar or slightly higher than the values found in the oils extracted under increasing oxygen flow. These results seem to be in contrast with what has been widely reported in the literature. However, several studies showed that the oxidative phenomena during malaxation increased as a function of oxygen availability [23,42,62,63,64]. On the other hand, the results obtained seem to agree with Sánchez-Ortiz et al. [65], who reported slightly higher peroxide values in oils extracted with addition of 20% of O2 during crushing, compared to oils from olives crushed under 60% of O2 in the cv Arbequina and Picual cultivars, while the opposite was observed for the cv. Blanqueta [66].

### 3.2. Phenolic Compounds

Table 2 shows the phenolic composition of the oils produced by adding different concentrations of oxygen during the crushing phase. For the two cultivars, the addition of different concentrations of oxygen (particularly 0.4 and 0.8 L/min) during the crushing phase resulted in a significant decrease in the total phenol concentration (*p* < 0.05) compared with the control test.

In both cultivars, the maximum decrease in total phenolic concentration was detected in the virgin olive oils produced by adding 0.8 L/min of oxygen during crushing. Thus, in the case of Ogliarola, the total concentration of phenols decreased 13% with respect to the control oil, from 645 mg/kg to 562 mg/kg. In addition, the phenolic compounds that are more influenced by the addition of oxygen in crushing are those with higher antioxidant activity, which are the oleuropein derivates [67,68]. Thus, the sum of oleuropein derivatives (hydroxytyrosol, oleacein, and oleuropein aglycon) decreased from 489 mg/kg (control oil) to 419 mg/kg in the case of 0.8 L/m of oxygen addition (Table 2). However, oleacein and oleuropein aglycone underwent a reduction of 14% and 16%, respectively, in their concentration. The reduction in the concentration of oleuropein derivatives can be related to an enzyme oxidation activity promoted by peroxidase and polyphenoloxidase, which use oxygen as a co-factor [69,70,71,72]. Furthermore, for the test adding 0.8 L/min of oxygen, a similar concentration decrease of the ligstroside derivates was detected, including tyrosol, oleocanthal, and ligstroside aglycon. This decrease accounted for 14% of the difference compared with the concentration of ligstroside derivatives in the control test, mainly due to the reduction of the concentration of oleocanthal (17%). On the contrary, the concentration of lignans did not show any significant variation.

When 0.4 L/min of oxygen was added, the concentration of total phenols underwent a 5% reduction, from 645 mg/kg in the control test to 611 mg/kg. In this case, the higher effect of reduction was also detected for the derivates of oleuropein and ligstroside. For example, a decrease of 5%, 10%, and 7% was observed for oleacein, oleuropein aglycon, and oleocanthal, respectively, in comparison with the control test. Finally, the treatment with 0.2 (L/min) of oxygen during crushing led to a non-significant reduction of only 2% in the total concentration of phenols.

The data for Coratina oils shows the same trend as the oil from the Ogliarola cultivar. Thus, the sum of total phenols showed its maximum value in the control test with no oxygen added. In Coratina oils, the maximum decrease of phenols was also observed with the addition of 0.8 (L/min) of oxygen. This oxygen treatment resulted in a significant (*p* < 0.05) decrease of 31% in total phenol concentration, from 856 mg/kg for the control test to 591 mg/kg for the test with 0.8 (L/min) of oxygen addition. This reduction percentage was higher than that observed in the Ogliarola cultivar (13%). The most relevant effect of oxygen addition was also identified for the oleuroprein derivates, especially for oleacein (52% reduction percentage). For the other concentrations of oxygen addition, a significant decrease of total phenolic compounds in comparison with the control test was observed: 11% and 2% of concentration decrease, respectively, for 0.4 L/min and 0.2 L/min of oxygen addition. The concentration of oleacein in the control test (555 mg/kg) underwent a reduction of 15% and 5% when oxygen was added at 0.4 L/min (472 mg/kg) and 0.2 L/min (527 mg/kg), respectively. On the contrary, an increase in the concentration of hydroxytyrosol and tyrosol was registered, although this change was not identified as significant. Derivates from ligstorside and lignans did not show significant variations for all the oils produced from Coratina olives, which contrasted with the results obtained with Ogliarola oils (Table 2).

The results obtained agree with Sánchez-Ortiz et al. [65], who detected a higher loss of phenols, particularly for oleuropein derivates, when a higher amount of oxygen (60%) in the crushing phase was applied. In that study, it was also possible to identify the cultivar influence on the oxidation of the phenolic compounds. However, the oxidation detected was more relevant in the oils coming from olives with higher concentrations of phenols, as was observed in the present study for oils from Coratina olives.

The results obtained agree with previous works focused on the malaxation phase and the role of oxygen in modifying the oil composition. Thus, Selvaggini et al. [41] detected that the oxygen added during the malaxation phase also reduced the quantity of phenols in oils due to the activities of the enzymes involved in the oxidation process of phenols. In that work, it was hypothesized that peroxydase (POD) and polyphenoloxidase (PPO) performed phenol oxidation due to the catalysis of the reaction in the presence of oxygen [23,73]. The reduction of phenols was also observed by El Riachy et al. [73], who reported that oleacein and oleuropein aglycone were considered to be the preferred choice in terms of substrate for activity in oxidative reactions carried out by PPO and POD. In the present study, the higher concentration of ligstroside derivatives detected in all the Coratina virgin olive oils made with the addition of oxygen during crushing, compared with the control test, seems to confirm the hypothesis above.

The observed effect of oxygen is supported by previous studies in which oxygen consumption and reactivity were measured during malaxation. In these studies, the oxidation activity of the enzymes that use oxygen as a co-factor was also evidenced. Thus, Amirante et al. [34], referring to the malaxer’s head space gas composition, showed that there is a rapid decrease in oxygen concentration, followed by a gradual reduction of its consumption inside the malaxer. The initial oxygen loss was attributed to the enzyme oxidation activities of PPO, POD, and LOX. Then, only in a second moment, the consumption of oxygen due to the cellular respiration process during all the rest of the malaxing phase can take place. Although there are not similar studies carried out during crushing, oxygen consumption can be due to analogous processes. Concerning CO2, in all the cases analyzed by Amirante et al. [34], any significant increase of this gas was only observed after the first eight minutes of malaxation. Thus, it is possible to hypothesize that the increase in oxygen concentration during the crushing phase can also promote the activity of PPO and POD with an effect on the phenol composition of the virgin olive oil, according to the data shown in Table 2. Nevertheless, considering that virgin olive oil phenols are first-type antioxidants, their higher concentration can be related to their antioxidant activity to avoid lipid oxidation. The protective effect of the phenolic compounds on the lipid matrix is confirmed by the non-significant variation of the quality parameters when oxygen is applied.

The different effects observed in the individual phenols can also be related to their antioxidant activity. Referring to this, several studies demonstrated that ortho-di-phenols such as hydroxytyrosol, oleacein 3,4-DHPEPA-EDA, and oleuropein aglycone are the most reactive substances and have the maximum antioxidant power, especially if compared with derivates of ligstroside and lignans [67,68]. The results of this work confirm the different reactivity of these phenols reported in these studies.

### 3.3. Volatile Compounds

Table 3 shows the data on volatile compounds detected in the virgin olive oils produced at different concentrations of oxygen added during the crushing phase. These data include the concentration of aldehydes, alcohols, esters, and ketones. From these compounds, aldehydes represent a relevant cluster in extra virgin olive oils because some of them produced by the LOX pathway are responsible for the green fruitiness of the oil. The micro-oxygenation seems to have a relevant effect from the point of view of the production of volatile compounds in the virgin olive oils obtained in the present work.

Additionally, virgin olive oils obtained by the addition of oxygen during the crushing phase showed significant variation (*p* < 0.05) in the composition of volatile compounds that come from LOX activity compared to the control test (no oxygen added). The test carried out under an oxygen flow of 0.2 L/min showed the highest concentration of aldehydes compared with all the other tests. This trend was detected in the oils from the two cultivars, with 19% and 8% of them increasing for Ogliarola and Coratina cultivar, respectively.

The virgin olive oil produced by the Ogliarola cultivar with a 0.2 L/min addition of oxygen showed a sum of C5 and C6 aldehydes of 21,146 µg/kg, which was significantly higher compared with the value of the control test (17,798 µg/kg) (Table 3). The most relevant increase was detected for (E)-2-hexenal, this compound being strictly related to “green grass” and “green fruity” aromas [4,74,75,76,77,78]. Unlike aldehydes, esters and alcohols showed a decreasing trend. Then, a significant decrease (*p* < 0.05) was detected for these two clusters of compounds, expressed as a decreasing of the sum of the C5 and C6 alcohols and, for the esters, a decrease of hexyl acetate and cis-3-hexenyl acetate. The lower concentration of these compounds may have an effect on the sensory profiling of the oils, given the reported sensory impact of alcohols and esters [78,79,80].

The oils obtained using an addition of 0.4 L/min of oxygen have a higher concentration of aldehydes (18,059 µg/kg) compared with the control oil (Table 3). However, this increase in concentration was only significant for (E)-2-hexenal. Regarding alcohols and esters, the oxygen under this flow reduced the concentration of these compounds by a significant amount. In the case of alcohols, it was also relevant to reduce some individual compounds by 20%. That was the case with 1-penten-3-ol, (E)-2-penten-1-ol, (Z)-2-penten-1-ol, 1-hexanol, (E)-2-hexen-1-ol, (Z)-2-hexen-1-ol, (E)-3-hexen-1-ol, and (Z)-3-hexen-1-ol.

The production of oil under an oxygen addition of 0.8 L/min during crushing resulted in the lowest mean concentration for all the clusters of volatile compounds (Table 3). However, the concentrations were significantly different from those in the control oil in the case of alcohols and esters. The concentration of alcohols showed the most relevant reduction (1407 µg/kg compared with 2057 µg/kg).

Similar results were observed in the oils from the Coratina cultivar. These oils also showed a significant increase in the sum of aldehydes, especially when oxygen was added at 0.2 L/min. Regarding the concentrations for each cluster, the values were different between the two cultivars (Table 3), thus pointing out that the oxygen effect was strongly modulated by the cultivar effect. The differences found in the volatile concentrations are explained by the particular enzyme activity in the volatile compound production (LOX) associated with each cultivar [78,81,82]. On the other hand, the enzyme activity is also modulated by the availability of oxygen during crushing, considering that it is a co-substrate of some enzymes that are involved in the LOX pathway.

The results confirm that, under the studied conditions, the best concentration of oxygen addition during the crushing phase was 0.2 L/min, considering the rise of aldehydes, particularly (E)-2-hexenal, and the effect on the other volatile compounds. In order to check if there is a better concentration of oxygen addition while also considering the cultivar and the extraction technology, it should be useful to make a sort of calibration line near this value. Considering the low quantity of oxygen used, and the interesting results also for sensory profiling, it could be useful in the future to have a recommended quantity of oxygen for each cultivar at a fixed condition of extraction technology. On the other hand, this study corroborates the importance of controlling oxygen availability in any research focused on the evaluation of different technologies of virgin olive oil extraction. Other studies also evidenced the effect of oxygen on volatile composition [23,83], although these studies focused on the oxygen availability during malaxation while the present work is focused on the crushing phase. The behavior of the enzymes of the lipoxygenase pathway is greatly affected during the crushing phase, where the conditions for the enzyme’s activity are different compared with malaxation in terms of both activity time and oxygen availability. On the other hand, in the aforementioned works, the procedure for modulating oxygen availability is different compared with the present works. Thus, for example, in one of those works [83], oxygen is reduced below the atmospheric concentration by insufflating nitrogen. In the other work [84], oxygen was directly applied to the olive paste during malaxation, although at a higher pressure (0–100 Kpa), and the effect of the volatile compounds greatly depended on the pressure applied. Due to the fact that the crushing phase is a continuous step in production, unlike malaxation, which is discontinuous, it was also expected that the results would not be comparable with those of the works on malaxation.

### 3.4. Sensory Analysis

In order to determine some differences related to the use of oxygen during the crushing phase and define the overall sensory profile of virgin olive oil samples from the cultivars under study, these samples were subjected to sensory analysis by 9 panelists. The evaluation was conducted using a special quantitative descriptive analysis form in which expert tasters reported the intensity of the various descriptors on unstructured scales.

In addition, different attributes were evaluated, concerning four aspects of visual evaluation (“deep green”, “moss green”, “yellow-green”, and “yellow”), eight related to the olfactory characteristics (“fruity”, “herbaceous”, “artichoke”, “hay”, “green apple”, “floral”, “tomato”, and “almond”), and four related to the gustatory-retro-olfactory attributes (“fatty”, “sweet”, “bitter”, and “pungent”). Moreover, off-flavors (“earthy”, “winey-vinegary”, “rancid”, and “fusty”) were also included in the evaluation sheet.

The sensory analyses carried out on the virgin olive oil samples confirmed the sensory impact of the chemical changes described above. Thus, the addition of oxygen during the crushing phase modified the sensory profile of the oils in accordance with the observed changes in phenols and volatile compounds.

**Figure 1:**
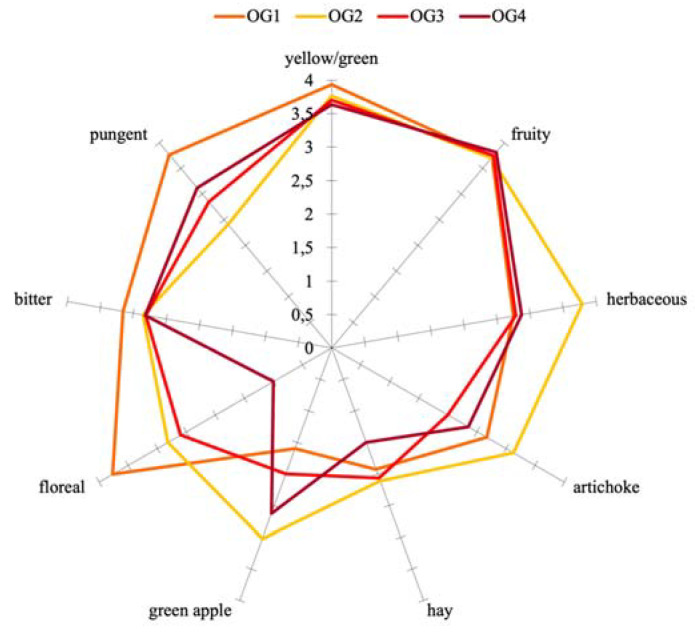
Sensory profile of Ogliarola olive oil obtained by adding three different concentrations of oxygen (0.2 L/min for OG2, 0.4 L/min for OG3, and 0.8 L/min for OG4) during crushing phase in comparison with control test (no oxygen added for OG1)

**Figure 2:**
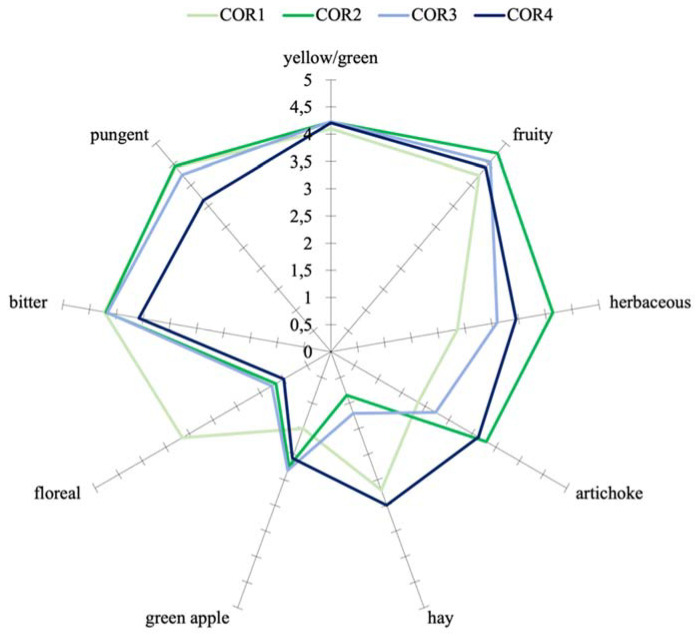
Sensory profile of cv. Coratina olive oil obtained by adding three different concentrations of oxygen (0.2 L/min for COR2, 0.4 L/min for COR3, and 0.8 L/min for COR4) during crushing phase in comparison with control test (no oxygen added for COR1)

Furthermore, from the sensory profiles of the virgin olive oils shown in Figure 1 and Figure 2, the attributes “fruity”, “pungent”, and “bitter” were clearly detected by the panelists (median > 4 in all the samples). None of the oils produced in this experiment presented sensory defects.

The availability of oxygen concentrations at increasing levels during crushing had an impact on sensory descriptors in all the samples tested compared to the virgin olive oils obtained by traditional processing (the control sample), although this effect was strongly influenced by the cultivar. In particular, the profiles of Ogliarola oils showed significant differences (*p* < 0.05) for the attribute “pungent”, while those of Coratina oils showed significant differences for the “artichoke”, “bitter”, and “pungent” attributes. The explanation of the sensory notes by the volatile composition also depended on the cultivar. Thus, for example, the herbaceous sensory note reported in the Ogliarola samples seems to be influenced by the concentration of (E)-2-hexenal (Table 3), and the sample presenting a higher intensity of this sensory note also showed a higher concentration of this aldehyde. In the case of Coratina oils, however, the influence of (E)-2-hexenal on the herbaceous perception seems to be less evident.

**Figure 3:**
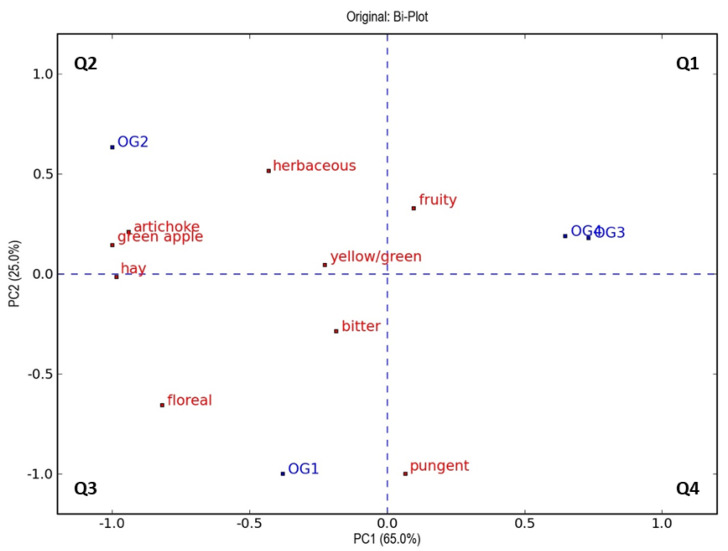
Representation of objects and variables (bi-plot) on the plane of the two principal components (PCA) relating to the results obtained through sensory evaluations of all virgin olive oils of cv. Ogliarola (objects) and attributes (variables) evaluated at the panel test. The variables and objects are marked in red and blue, respectively. The four quadrants are coded as Q1–Q4

**Figure 4:**
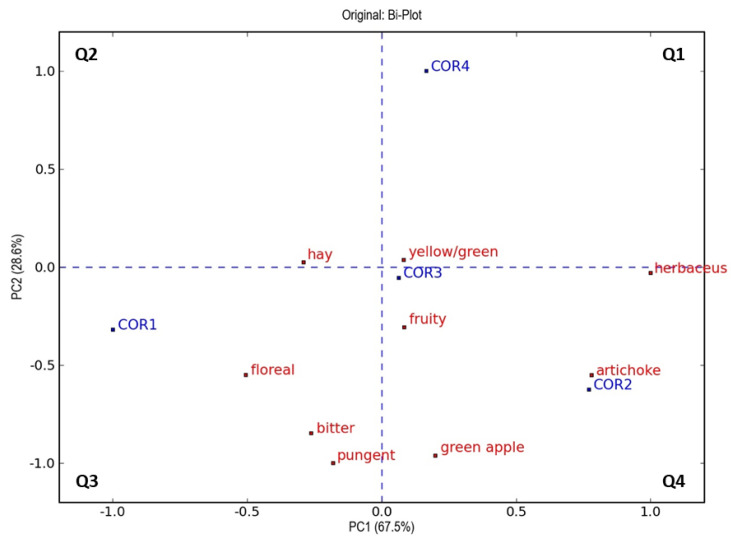
Representation of objects and variables (bi-plot) on the plane of the two principal components (PCA) relating to the results obtained through sensory evaluations of all virgin olive oils of cv. Coratina and the attributes evaluated. The variables and objects are marked in red and blue, respectively. The four quadrants are coded as Q1–Q4

In order to identify changes in the sensory profile from a multivariate perspective, principal component analysis (PCA) was carried out with the scores provided by the panelists. PCA plots, shown in Figure 3 and Figure 4, underline that the oils obtained with oxygenation treatment are characterized by a higher intensity of some “green” sensory notes, such as “fruity”, “herbaceous”, “artichoke”, and “green apple”, compared to control samples [4,74,81]. This effect is more pronounced in oils extracted under oxygen flow at 0.2 L/min, which presented a higher content of volatile compounds derived from the lipoxygenase pathway (C5 and C6 aldehydes, either saturated or unsaturated), contributing to “green” attributes [84] in both cultivars (Table 3).

On the other hand, the intensity of “bitter” and “pungent” sensory notes tends to decrease as the concentration of oxygen applied during crushing increases, in parallel with the reduction of the phenolic compounds that are responsible for these attributes [85,86].

The distribution of the samples in the four quadrants allows us to describe them and highlight the possible dependencies as a function of the variables present in the same quadrants. The PCA model shown in Figure 3 explains 90.0% of the total variance with two principal components in the data (the first and second components explaining 65.0% and 25.0%, respectively). From the bi-plot relating these components, a clear separation of samples emerges along the second component (from negative to positive score values) in relation to the oxygenation treatment/non-treatment applied, with the control sample characterized by negative score values (quadrant 3). The samples treated with increasing oxygen concentration were characterized by positive score values relative to principal component 2, and the sample treated with an oxygen addition of 0.2 L/min (OG2 in Figure 3) was plotted in a different quadrant (quadrant 2) compared to the other treated samples (quadrant 1). On the other hand, from the first component of the bi-plot (reading from negative to positive score values), it was also observed that oils were extracted differently according to the concentration of oxygen used. Then, samples OG1 and OG2 were characterized by negative values of principal component 1, while samples OG3 and OG4 were characterized by positive score values.

Additionally, from the bi-plot of this PCA model, it can be inferred that the location of the OG2 sample in the plot can be explained by the variables “green apple”, “artichoke”, “herbaceous”, and “hay”, while the control sample was related to the attributes “pungent” and “floreal.” This relationship was in accordance with the higher values of these sensory attributes for these two samples, as observed in Figure 1.

Similarly, another PCA model was built with all the variables obtained from the sensory analysis of Coratina virgin olive oils, shown in Figure 4. In this case, the two principal components explained 96.1% of the total variance of the data (67.5% for the first component and 28.6% for the second). The plot also highlighted how the principal discrimination of the objects is mainly due to the oxygenation treatment carried out, as shown by the reading along the second component (from negative to positive values). According to the plot, the COR2 sample was related to the attributes “artichoke” and “herbaceous”, which were characterized by higher intensity as observed in Figure 2. The plot also demonstrated that the oxygen treatment in COR4 (0.8 L/min of added oxygen) produced a different sensory profile, given that this sample was the only one with positive score values for principal component 2.

Furthermore, reading the PCA along the first component, it was observed a differentiation of the samples between those treated with oxygen during crushing (COR2, COR3, and COR4) and the control sample (COR1), the latter characterized by negative score values for this component (Figure 4). This difference was characterized by higher intensities of “hay” and “floral” in the control samples and a higher intensity of “yellow/green”, “fruity”, “green apple”, “herbaceous”, and “artichoke” in the treated samples. These results are in accordance with what was observed by the headspace analysis of the same samples, in which it was evident a higher concentration of volatile compounds such as 1-penten-3-ol, (E)-2-hexenol, (Z)-3-hexenol, and (Z)-3-hexenyl acetate, for the control oils and higher concentrations of (E)-2-hexenal for the treated oils, especially for COR2.

## 4. Conclusions

This study has shown the effect of oxygen supply during crushing on two different cultivars. The effect can be positive if an adequate addition flow is used to increase volatile compounds that provide positive attributes (e.g., green aroma), but with a minor effect on the concentration of phenolic compounds. In this work, the most adequate oxygen addition was shown to be 0.2 L/min in the two studied cultivars. However, all the oxygen addition permitted the production of virgin olive oil with all the legal quality parameters within the extra virgin olive oil category according to EU regulation. Thus, the oxygen addition can be used to modulate sensory characteristics, particularly from the point of view of aroma, while at the same time keeping the legal parameters under control. The cultivar effect was relevant, and the results showed the importance of enzyme activities in these cultivars in relation to the effect of adding oxygen during crushing. Maturity can also have an influence on the oxygen effect, although this factor would require a specific study. In addition to cultivar and maturity, given the wide range of phenol concentrations found in EVOOs, the study of the role of these compounds in optimizing oxygen load could also be addressed to better understand the oxygen influence on quality. Furthermore, the results also supported some observations carried out in other phases of the production (e.g., malaxation). This work supports the idea of finding new procedures to manage all the factors that affect virgin olive oil quality and composition during all the phases of production. Thus, this work also shows the importance of controlling all the production variables, including those that are not always controlled, such as oxygen, which can modify the quality of the final product according to the desired sensory characteristic. Furthermore, the production of fruitier and tastier extra virgin olive oils can improve the consumption of this product and promote different varieties. Especially for extra virgin olive oil, it is important to protect biodiversity in order to have a more resilient production. Moreover, these studies are important to improve the production system in general and to incorporate innovations that can be developed further.

## Figures and Tables

**Table 1:** Legal quality parameter of VOOs extracted with different values of oxygen flow during the crushing phase

Parameters	*Ogliarola*				*Coratina*			
	OG1 (No O_2_ Supply)	OG2 (O_2_ 0.2 L/min)	OG3 (O_2_ 0.4 L/min)	OG4 (O_2_ 0.8 L/min)	COR1 (No O_2_ Supply)	COR2 (O_2_ 0.2 L/min)	COR3 (O_2_ 0.4 L/min)	COR4 (O_2_ 0.8 L/min)
Acidity (% oleic ac.)	0.35 ± 0.04 a	0.31 ± 0.00 a	0.33 ± 0.00 a	0.32 ± 0.01 a	0.22 ± 0.01 a	0.25 ± 0.02 a	0.22 ± 0.01 a	0.23 ± 0.02 a
Peroxide value (meq O_2_/kg of oil)	7.6 ± 0.6 a	7 ± 0.3 a	6.8 ± 0.1 a	7.5 ± 0.1 a	4.65 ± 0.8 a	4.15 ± 0.07 a	4.65 ± 0.2 a	4.2 ± 0.3 a
K232	1.79 ± 0.00 b	1.78 ± 0.02 b	1.71 ± 0.03 c	1.84 ± 0.06 a	1.62 ± 0.06 a	1.56 ± 0.04 a	1.61 ± 0.02 a	1.61 ± 0.08 a
K270	0.13 ± 0.00 a	0.15 ± 0.01 a	0.14 ± 0.01 a	0.13 ± 0.00 a	0.15 ± 0.00 a	0.16 ± 0.01 a	0.16 ± 0.01 a	0.16 ± 0.01 a

Note: Values are the mean of two determinations in two independent trials ± the standard deviation. For each different cultivar, different letters (a–c) in the row indicate that results are statistically different from one another (*p* < 0.05). The ΔK was lower than 0.01 in all cases

**Table 2:** Phenolic composition (mg/kg) of VOOs extracted with different values of oxygen flow during the crushing phase

Phenolic Compounds	*Ogliarola*				*Coratina*			
	OG1	OG2	OG3	OG4	COR1	COR2	COR3	COR4
	(No O_2_ Supply)	(O_2_ 0.2 L/min)	(O_2_ 0.4 L/min)	(O_2_ 0.8 L/min)	(No O_2_ Supply)	(O_2_ 0.2 L/min)	(O_2_ 0.4 L/min)	(O_2_ 0.8 L/min)
Hydroxytyrosol	1.6 ± 0.1 b	3.2 ± 0.8 a	1.7 ± 0 b	1.7 ± 0.2 b	1 ± 0.0 b	1.3 ± 0.1 a	1.2 ± 0.0 a	1 ± 0.1 b
Tyrosol	4.4 ± 0.6 a	4.2 ± 0.3 a	4.3 ± 0.2 a	4.3 ± 0.0 a	2 ± 0.1 b	2.4 ± 0.2 ab	2.5 ± 0.3 a	2 ± 0.0 b
Vanillic acid	0.4 ± 0.1 a	0.3 ± 0.0 a	0.3 ± 0.0 a	0.4 ± 0.0 a	0.5 ± 0.1 ab	0.6 ± 0.1 a	0.6 ± 0.1 a	0.4 ± 0.0 b
*p*-Coumaric acid	0.3 ± 0.0 a	0.3 ± 0.0 a	0.3 ± 0.0 a	0.3 ± 0.0 a	n.d.	n.d.	n.d.	n.d.
Oleacein	384.1 ± 20.4 a	375.4 ± 23.2 a	364.8 ± 31.4 a	330.4 ± 15.6 a	555.3 ± 8.6 a	527.1 ± 3.2 a	472.3 ± 6.1 b	268.1 ± 37.8 c
Oleocanthal	71.1 ± 4.4 a	69.1 ± 8.8 a	66 ± 5.2 a	59.8 ± 2.9 a	105 ± 5.8 a	106.3 ± 0.7 a	105.3 ± 5.7 a	107.4 ± 3.1 a
(+)-1-acetoxypinoresinol	42.6 ± 1.1 a	41 ± 1.6 a	42.6 ± 1.7 a	41.6 ± 0.3 a	33.4 ± 0.2 a	34.2 ± 0.3 a	33.5 ± 1 a	34.3 ± 3.3 a
(+)-pinoresinol	19.1 ± 0.7 a	19.4 ± 0.8 a	19.8 ± 2.2 a	19.5 ± 0.7 a	15.2 ± 0.3 a	15.4 ± 0.4 a	15 ± 0.7 a	13.9 ± 1.6 a
Oleuropein aglycone	103.6 ± 5.5 a	101.1 ± 6.4 a	93.5 ± 4.4 ab	87 ± 0.2 b	126.5 ± 7.0 b	128.5 ± 2.3 b	112.7 ± 1.4 b	146.5 ± 10.4 a
Ligstroside aglycone	17.5 ± 1.6 a	16.4 ± 1.0 a	17.6 ± 0.4 a	16.7 ± 0.8 a	17.4 ± 2.2 b	21.3 ± 1.7 ab	22.2 ± 1.3 a	17.6 ± 0.6 b
Sum of Oleuropein derivatives	489.3 ± 21.1 a	459.9 ± 24.1 ab	479.6 ± 31.7 ab	419 ± 15.6 b	682.9 ± 11.1 a	657 ± 3.9 a	586.2 ± 6.2 b	415.6 ± 39.2 c
Sum of ligstroside derivatives	582.3 ± 4.7 a	547.8 ± 8.8 a	569.4 ± 5.2 b	499.9 ± 3.0 c	124.4 ± 6.2 a	127 ± 3.1 a	130 ± 5.9 a	129.9 ± 1.8 a
Sum of lignans derivatives	61.7 ± 1.3 a	62.4 ± 1.8 a	60.4 ± 2.7 a	61.1 ± 0.8 a	48.6 ± 0.4 a	49.6 ± 0.5 a	48.5 ± 1.2 a	48.3 ± 3.7 a
Total phenol	644.8 ± 22.3 a	630.4 ± 26.5 a	610.8 ± 32.5 ab	561.7 ± 15.9 b	856.4 ± 14.6 a	837.1 ± 4.9 a	765.2 ± 8.8 b	591.2 ± 40.8 c

Note: Results are the mean of two determinations in two independent experiments ± the standard deviation. Included in the sum of oleuropein derivatives are hydroxytyrosol. oleacein and oleuropein aglycone. in the sum of ligustroside derivatives tyrosol. oleochanthal and ligstroside aglycon. in that of lignans (+)-1-acetoxypinoresinol and (+)-pinoresinol. For each different cultivar, different lowercase letters (a, b, c) indicate that the results are statistically different from one another (*p* < 0.05). n.d., non detected

**Table 3:** Volatile composition (µg/kg) of VOOs extracted with different values of oxygen flow during the crushing phase

Volatile Compounds	*Ogliarola*				*Coratina*			
	OG1 (No O_2_ Supply)	OG2 (O_2_ 0.2 L/min)	OG3 (O_2_ 0.4 L/min)	OG4 (O_2_ 0.8 L/min)	COR1 (No O_2_ Supply)	COR2 (O_2_ 0.2 L/min)	COR3 (O_2_ 0.4 L/min)	COR4 (O_2_ 0.8 L/min)
*Aldehydes*								
Pentanal	8 ± 1 a	8 ± 0 a	6 ± 1 a	10 ± 4 a	13 ± 0 a	17 ± 0 a	18 ± 4 a	12 ± 11 a
(*E*)-2-Pentenal	44 ± 2 a	47 ± 3 a	38 ± 2 b	36 ± 1 c	60 ± 9 a	54 ± 3 a	55 ± 1 a	35 ± 24 a
Hexanal	908 ± 20 a	1009 ± 97 a	820 ± 14 b	774 ± 19 c	2298 ± 7 a	2051 ± 144 b	1954 ± 39 b	1960 ± 111 b
(*Z*)-3-Hexenal	n.d.	n.d.	n.d.	n.d.	n.d.	n.d.	n.d.	n.d.
(*E*)-2-Hexenal	16,772 ± 444 c	20,007 ± 537 a	17,137 ± 239 b	16,470 ± 386 d	44,408 ± 1906 b	48,329 ± 599 a	45,871 ± 1211 a	39,504 ± 1545 c
(*E,E*)-2,4-Hexadienal	66 ± 1 a	75 ± 7 a	58 ± 3 b	61 ± 3 ab	172 ± 1 a	158 ± 4 ab	162 ± 0 a	140 ± 16 b
Sum of aldehydes at C_5_ and C_6_	17,798 ± 444 b	21,146 ± 545 a	18,059 ± 239 b	17,351 ± 386 b	46,950 ± 1906 b	50,609 ± 616 a	48,059 ± 1212 a	41,651 ± 1549 b
*Alcohols*								
1-Pentanol	27 ± 4 ab	31 ± 0 a	23 ± 1 b	23 ± 1 b	41 ± 0 a	42 ± 0 a	41 ± 1 a	37 ± 5 a
1-Penten-3-ol	210 ± 14 a	170 ± 11 b	172 ± 8 b	172 ± 5 b	621 ± 54 a	491 ± 23 b	509 ± 2 b	484 ± 13 b
(*E*)-2-Penten-1-ol	28 ± 1 b	34 ± 2 a	24 ± 1 c	23 ± 0 c	57 ± 10 a	48 ± 2 a	47 ± 1 a	41 ± 9 a
(*Z*)-2-Penten-1-ol	201 ± 15 a	222 ±24 a	162 ± 2 b	162 ± 0 b	661 ± 105 a	559 ± 21 a	565 ± 9 a	538 ± 25 a
1-Hexanol	748 ± 55 a	577 ± 21 b	547 ± 46 b	514 ± 3 b	649 ± 113 a	497 ± 33 b	596 ± 19 b	463 ± 47 b
(*E*)-2-Hexen-1-ol	669 ± 37 a	518 ± 29 b	401 ± 4 c	392 ± 8 d	1280 ± 38 a	977 ± 41 c	1152 ± 22 b	935 ± 16 c
(*Z*)-2-Hexen-1-ol	0 ± 0	0 ± 0	0 ± 0	0 ± 0	0 ± 0	0 ± 0	0 ± 0	0 ± 0
(*E*)-3-Hexen-1-ol	15 ± 4 a	11 ± 1 a	10 ± 0 a	10 ± 1 a	0 ± 0	0 ± 0	5 ± 0	0 ± 0
(*Z*)-3-Hexen-1-ol	159 ± 25 a	118 ± 15 b	106 ± 9 b	112 ± 7 b	373 ± 37 a	286 ± 9 b	298 ± 22 b	237 ± 29 b
Sum of alcohols at C_5_ and C_6_	2057 ± 74 a	1681 ± 47 b	1445 ± 48 c	1407 ± 12 c	3683 ± 172 a	2898 ± 62 c	3212 ± 39 b	2735 ± 65 c
*Esters*								
Hexyl acetate	43 ± 5 a	27 ± 1 b	25 ± 1 b	27 ± 4 b	0 ± 0	0 ± 0	0 ± 0	0 ± 0
(*Z*)-3-Hexenyl acetate	31 ± 4 a	19 ± 1 b	16 ± 0 b	18 ± 1 b	6 ± 1 a	4 ± 0 b	4 ± 0 b	4 ± 0 b
Sum of esters at C_6_	74 ± 6 a	46 ± 2 b	42 ± 1 b	45 ± 4 b	6 ± 1 a	4 ± 0 b	4 ± 0 b	4 ± 0 b

Note: The data are the mean values of two independent extractions, ± standard deviation. For each different cultivar, the values in each row having different letters (a, b, c, d) are significantly different from one another. n.d., non detected

## Data Availability

The data used to support the findings of this study can be made available by the corresponding author upon request.
